# Molecular Phylogeny and Historical Biogeography of the Neotropical Swarm-Founding Social Wasp Genus *Synoeca* (Hymenoptera: Vespidae)

**DOI:** 10.1371/journal.pone.0119151

**Published:** 2015-03-04

**Authors:** Rodolpho Santos Telles Menezes, Seán Gary Brady, Antônio Freire Carvalho, Marco Antonio Del Lama, Marco Antônio Costa

**Affiliations:** 1 Departamento de Ciências Biológicas, Universidade Estadual de Santa Cruz, Ilhéus, Bahia, Brazil; 2 Department of Entomology, National Museum of Natural History, Smithsonian Institution, Washington, DC, United States of America; 3 Departamento de Genética e Evolução, Universidade Federal de São Carlos, São Carlos, São Paulo, Brazil

## Abstract

The Neotropical Region harbors high biodiversity and many studies on mammals, reptiles, amphibians and avifauna have investigated the causes for this pattern. However, there is a paucity of such studies that focus on Neotropical insect groups. *Synoeca* de Saussure, 1852 is a Neotropical swarm-founding social wasp genus with five described species that is broadly and conspicuously distributed throughout the Neotropics. Here, we infer the phylogenetic relationships, diversification times, and historical biogeography of *Synoeca* species. We also investigate samples of the disjoint populations of *S*. *septentrionalis* that occur in both northwestern parts of South America through Central American and the Brazilian Atlantic rainforests. Our results showed that the interspecific relationships for *Synoeca* could be described as follows: (*S*. *chalibea *+ *S*. *virginea*) + (*S*. *cyanea* + (*S*. *septentrionalis*/*S*. *surinama*)). Notably, samples of *S*. *septentrionalis* and *S*. *surinama* collected in the Atlantic Forest were interrelated and may be the result of incomplete lineage sorting and/or mitochondrial introgression among them. Our Bayesian divergence dating analysis revealed recent Plio-Pleistocene diversification in *Synoeca*. Moreover, our biogeographical analysis suggested an Amazonian origin of *Synoeca*, with three main dispersal events subsequently occurring during the Plio-Pleistocene.

## Introduction

The Neotropical Region is one of the most important biodiversity hotspots in the world, harboring more than half of the Earth’s remaining rainforests [1]. Recent studies documenting the diversification history of the Neotropical biota have elucidated both temporal and spatial biogeographic patterns, resulting in proposals that diversification events in the South American biota have been driven by the uplift of the Andes, marine incursions, or Pleistocene climate changes [2–5]. In addition, studies involving mammals and birds have allowed the inference of historical connections between the morphoclimatic domains of the South America Amazonian rainforest (AM) and the Atlantic Forest (AF) (e.g., [6–7]). Currently these two forests are separated by a dry corridor of open vegetation [8], also called the ‘Dry Diagonal’, that encompasses the Argentinean and Paraguayan Chaco, the Caatinga in northeastern Brazil, and the central Brazilian Cerrado [6,8].

*Synoeca* de Saussure, 1852 (Hymenoptera: Vespidae: Epiponini) is a Neotropical swarm-founding social wasp genus with five described species: *Synoeca chalibea* de Saussure, 1852; *Synoeca virginea* (Fabricius, 1804); *Synoeca surinama* (Linnaeus, 1767); *Synoeca septentrionalis* Richards, 1978; and *Synoeca cyanea* (Fabricius, 1775). Species in this genus display vigorous defense of their nests accompanied by painful sting. Their nests are directly attached to the substrate and have a convex envelope that may present regular transverse corrugations resembling the back of an armadillo (Dasypodidae), and so are known in many parts of Latin America as “cachicamas”, “armadillas”, “conchajonas”, “carachupa”, “caba-tatu” or “marimbondo-tatu” [9–14]. *Synoeca* species have been the focus of many studies about their behavior [10, 12–15], caste differentiation [16–17] and nest architecture [11, 18]. They are broadly distributed in the Neotropical region, ranging from central Mexico down to northern Argentina [9]. *S*ynoeca *virginea* is restricted to AM; *S*. *chalibea* also occurs in AM, but can be found in Costa Rica and Panama as well. *Synoeca surinama* is widely distributed in South American rainforests and, conspicuously, in the Brazilian savannah (i.e., the Cerrado) as well. *Synoeca septentrionalis* is distributed from northwestern parts of South America through Central America and into central Mexico; however, recently specimens have been found in the AF. *Synoeca cyanea* is the only species that is restricted to the eastern portion of South America (ESA) [9, 19].

Several previous studies have sought to understand the phylogenetic relationships among *Synoeca* species [20–22]. These analyses used only morphological characters and supported the monophyly of the genus, with the following relationships among the species: *S*. *chalibea* + (*S*. *virginea* + (*S*. *septentrionalis* + (*S*. *surinama* + *S*. *cyanea*))) [20, 22]. However, species of *Synoeca* exhibit wide morphological variation, as indicated by Cely and Sarmiento [21] and the presence of multiple synonyms for species [9]. Moreover, the recent record of colonies of *S*. *septentrionalis* in the AF [19] raises questions about the taxonomy and evolutionary history of the group.

Here we infer the first molecular phylogeny for *Synoeca* species using mitochondrial and nuclear DNA sequences. We also provide new observations of morphological structures that can help diagnose some species. Furthermore, we investigate the status of the disjoint populations of *S*. *septentrionalis*. Bayesian divergence dating analysis calibrated with an *a priori* mutation rate of insect mitochondrial DNA was used to estimate a timescale for diversification events in the genus. We also provide a Bayesian analysis of historical biogeography that suggests an Amazonian origin for *Synoeca*.

## Material and Methods

### Taxon sampling

**Fig 1:**
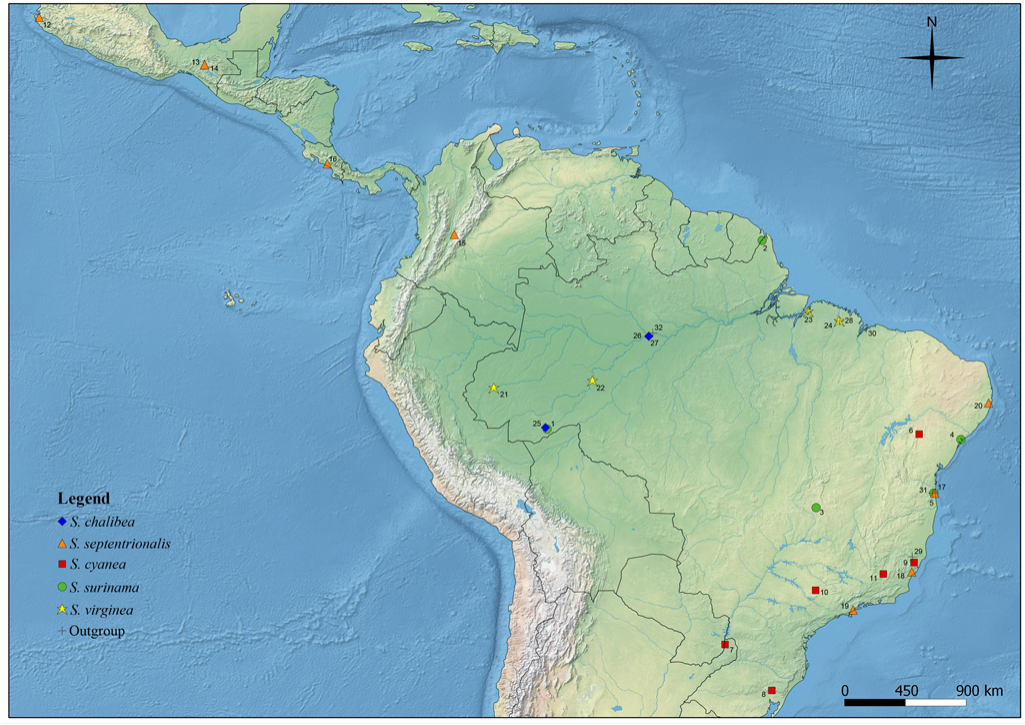
Map showing collection sites. The numbers refer to the sampling locality codes shown in Table 1.

Our sampling included specimens from multiple localities within known species distributions. Table 1 and Fig. 1 show the collection sites of specimens used in this study. The map was generated using the software Quantum-GIS v1.8.0 (Open Source Geospatial Foundation Project, Beaverton, OR, USA). All specimens were preserved in ethanol prior to the molecular analyses and vouchers are deposited in the entomological collections at the Universidade Estadual de Santa Cruz, Ilhéus, Bahia, Brazil and the National Museum of Natural History (USNM, Washington, DC). All necessary research permits for fieldwork and collection of samples by RSTM and AFC were issued by the Brazilian Institute for Biodiversity Conservation (ICMBio) recorded by SISBio (permit numbers 22180 and 5374523). Field studies did not involve endangered or protected species. The samples from localities below were donated from different researchers: Wanda, Misiones, Argentina: 25°58'55" S, 54°34'58" W; El Tuito, Jalisco, Mexico: 20°19'02" N, 105°19'42" W; Chiapas, Mexico: 16°48'05" N, 93°05'18" W; Pandi, Cundinamarca, Colombia: 4°;15'32" N, 74°36'35" W; Santa Rosa, Guanacaste, Costa Rica: 10°50'06" N, 85°42'12" W".

**Table 1:** Descriptive information for all taxa used in this phylogenetic study.

Species	Map ID	Locality	Coordinates	GenBank accession number			
				16S	COI	CytB	Wg
*Synoeca surinama	1	Rio Branco, Acre, Brazil	10°04'05" S, 67°45'03" W	KJ829620	KJ829539	KJ829569	KJ829595
*Synoeca surinama	2	Oiapoque, Amapá, Brazil	3°49'52" N, 51°50'22" W	KJ829621	KJ829540	KJ829570	KJ829596
*Synoeca surinama	3	Brasília, Distrito Federal, Brazil	15°52'28" S, 47°50'52" W	KJ829624	KJ829543	KJ829573	KJ829599
*Synoeca surinama	4	Nossa Senhora do Socorro, Sergipe, Brazil	10°50'30" S, 37°08'21" W	KJ829622	KJ829541	KJ829571	KJ829597
*Synoeca surinama	5	Ilhéus, Bahia, Brazil	14°48'10" S, 39°08'26" W	KJ829623	KJ829542	KJ829572	KJ829598
*Synoeca cyanea	6	Senhor do Bonfim, Bahia, Brazil	10°26'44" S, 40°13'23" W	KJ829635	KJ829553	KJ829584	KJ829608
*Synoeca cyanea*	7	Wanda, Misiones, Argentina	25°58'55" S, 54°34'58" W	-	-	KJ829588	-
*Synoeca cyanea	8	Nova Petrópolis, Rio Grande do Sul, Brazil	29°22'29" S, 51°07'30" W	KJ829638	KJ829556	KJ829587	KJ829611
*Synoeca cyanea	9	Santa Teresa, Espírito Santo, Brazil	19°55'22" S, 40°35'58" W	KJ829637	KJ829555	KJ829586	KJ829610
*Synoeca cyanea	10	São Carlos, São Paulo, Brazil	21°58'33" S, 47°52'57" W	KJ829636	KJ829554	KJ829585	KJ829609
*Synoeca cyanea	11	Viçosa, Minas Gerais, Brazil	20°46'29" S, 42°52'30" W	KJ829634	KJ829552	KJ829583	KJ829607
*Synoeca septentrionalis*	12	El tuito, Jalisco, México	20°19'02" N, 105°19'42" W	KJ829627	KJ829546	KJ829576	KJ829602
*Synoeca septentrionalis*	13	Chiapas, México	16°48'05" N, 93°05'18" W	KJ829631	KJ829550	KJ829580	KJ829605
*Synoeca septentrionalis*	14	Chiapas, México	16°48'05" N, 93°05'18" W	KJ829632	-	KJ829581	-
*Synoeca septentrionalis*	15	Pandi, Cundinamarca, Colombia	4°15'32" N, 74°36'35" W	KJ829630	KJ829549	KJ829579	KJ829604
*Synoeca septentrionalis*	16	Santa Rosa, Guanacaste, Costa Rica	10°50'06" N, 85°42'12" W	KJ829629	KJ829548	KJ829578	-
*Synoeca septentrionalis	17	Ilhéus, Bahia, Brazil	14°47'58" S, 39°04'36" W	KJ829628	KJ829547	KJ829577	KJ829603
*Synoeca septentrionalis	18	Alfredo Chaves, Espírito Santo, Brazil	20°37'33" S, 40°46'14" W	KJ829633	KJ829551	KJ829582	KJ829606
*Synoeca septentrionalis	19	Ubatuba, São Paulo, Brazil	23°26'13" S, 45°06'46" W	KJ829625	KJ829544	KJ829574	KJ829600
*Synoeca septentrionalis	20	Moreno, Pernambuco, Brazil	8°08'24" S, 35°04'41" W	KJ829626	KJ829545	KJ829575	KJ829601
*Synoeca virginea*	21	Ipixuna, Amazonas, Brazil	7°03'00" S, 71°41'38" W	-	-	KJ829566	KJ829592
*Synoeca virginea*	22	Canutama, Amazonas, Brazil	6°31'29" S, 64°23'47" W	KJ829618	-	KJ829567	KJ829593
*Synoeca virginea*	23	Belém, Pará, Brazil	1°26'25" S, 48°25'32" W	KJ829619	KJ829538	KJ829568	KJ829594
*Synoeca virginea*	24	Centro Novo do Maranhão, Maranhão, Brazil	2°08'17" S, 46°08'16" W	KJ829617	-	KJ829565	-
*Synoeca chalibea	25	Rio Branco, Acre, Brazil	9°57'31" S, 67°52'10" W	KJ829640	KJ829558	KJ829590	-
*Synoeca chalibea*	26	Iranduba, Amazonas, Brazil	3°15'08" S, 60°13'33" W	KJ829639	KJ829557	KJ829589	KJ829612
*Clypearia weyrauchi	27	Iranduba, Amazonas, Brazil	3°15'08" S, 60°13'33" W	KJ829644	KJ829563	-	KJ829616
*Asteloeca traili*	28	Centro Novo do Maranhão, Maranhão, Brazil	2°08'17" S, 46°08'16" W	KJ829645	KJ829564	-	-
*Metapolybia decorata	29	Colatina, Espírito Santo, Brazil	19°30'55" S, 40°43'21" W	-	KJ829559	-	KJ829613
*Metapolybia docilis	30	São Luiz, Maranhão, Brazil	2°39'05" S, 44°08'29" W	KJ829641	KJ829560	-	-
*Epipona media	31	Ilhéus, Bahia, Brazil	14°47'31" S, 39°12'52" W	KJ829643	KJ829562	KJ829591	KJ829615
*Epipona tatua	32	Manaus, Amazonas, Brazil	3°00'23" S, 59°56'19" W	KJ829642	KJ829561	-	KJ829614

An asterisk indicates specimens collected directly from nests.

### Species identification and morphological observations

**Fig 2:**
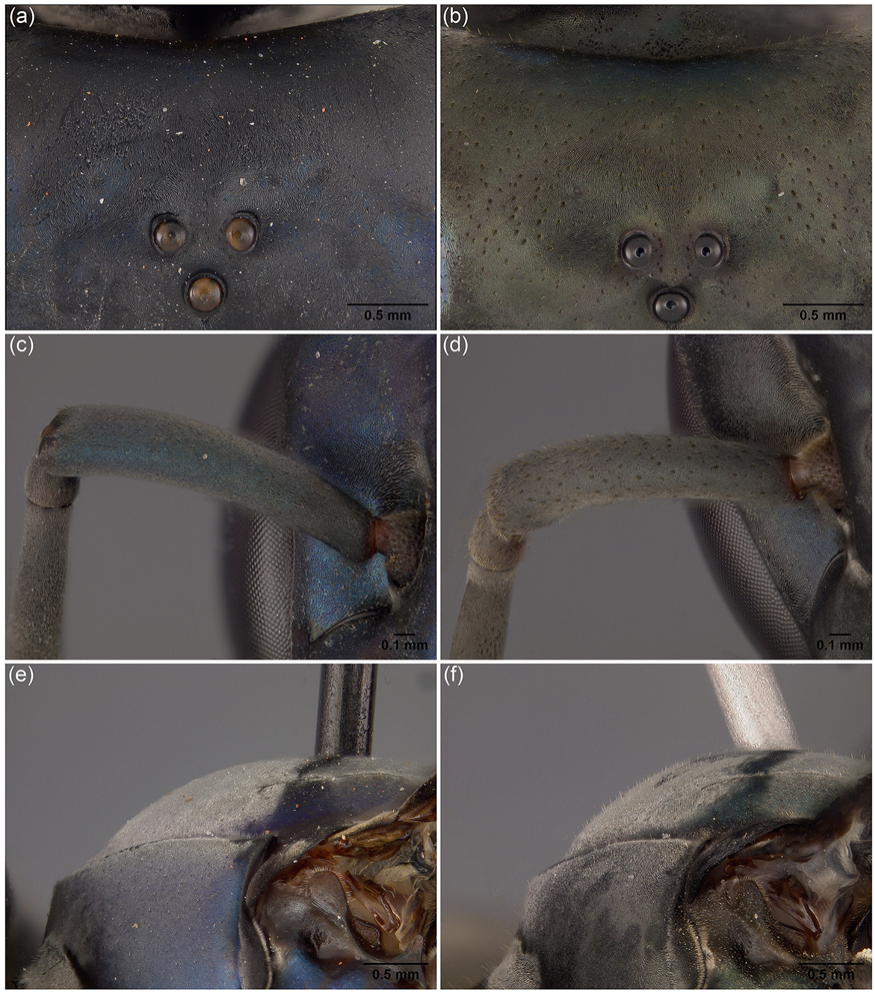
External morphological structures observed. View frontal of head, (a) *S*. *surinama* and (b) *S*. *septentrionalis*. View frontal of scape, (c) *S*. *cyanea* and (d) *S*. *septentrionalis*. Scutum in lateral view, (e) *S*. *surinama* and (f) *S*. *septentrionalis*.

Species identification was based on the keys provided by Richards [9], Andena et al. [20], and Cely and Sarmiento [21]. We observed additional external morphological structures which helped in identifying specimens, as follows: punctation on the head; erect setae on the scape; and erect setae on the scutum (Fig. 2). Images of specimens were generated using a JVC KY–F75U digital camera mounted on a Leica Z16 APO steromicroscope. All images were edited using Photoshop CS4 (Version 11.0) (Adobe Inc.).

### DNA extraction, amplification and sequencing

The mesosoma and/or hind legs were removed from each specimen and DNA was extracted using the phenol-chloroform method following the Han and McPheron protocol [23]. We amplified three mitochondrial (16S, cytochrome b, and cytochrome c oxidase I) and one nuclear (wingless) gene fragments by PCR using specific primers and amplification conditions (S1 Table). PCR products were purified using exonuclease I and shrimp alkaline phosphatase and directly sequenced in an ABI Prism 3730 (Applied Biosystems) sequencer (Laboratório de Biotecnologia da FCAV—UNESP de Jaboticabal, SP). PCR products were sequenced in both directions and sequence contigs were assembled using Sequencher 5.1 (Gene Code Corp., Ann Arbor, MI, USA). DNA sequences were aligned using Muscle 3.7 [24] (with default parameters) in MEGA 5.10 [25], with each of the four genes aligned separately. All sequences are deposited in GenBank and accession numbers are listed in Table 1.

### Phylogenetic analyses

We included DNA data from 26 *Synoeca* samples with each of the five *Synoeca* species represented by multiple collection localities. We used species from four other genera as outgroups. Most phylogenetic analyses were based on a concatenated data matrix (1829 base pairs) of the four gene fragments. The most appropriate model of nucleotide evolution and the best-fitting partitioning scheme were selected using PartitionFinder v1.1.1 [26] under the Bayesian information criterion (BIC) (Table 2). Phylogenetic inference was conducted by Bayesian inference (BI) performed using MrBayes v3.2.2 [27] and maximum likelihood (ML) performed using GARLI v2.0 [28]. BI was also performed on each of the three mitochondrial gene fragments (wingless contained little phylogenetic information) separately to examine potential conflicts in phylogenetic signal among genes. All BI analyses consisted of two independent runs of 50 million generations each with four chains (temp = 0.1) and sampled every 1000 generations. The burn-in, convergence, and stationarity were assessed using Tracer v1.5 [29]. We removed the first 20% of sampled generations and combined the remaining generations to produce the maximum credibility tree. We conducted 1000 ML bootstrap replicates in GARLI under the same partitions and nucleotide models as in BI. Trees from all analyses were visualized using FigTree v1.4.0 program [30].

**Table 2:** Data partitions and nucleotide substitution models suggested by PartitionFinder v1.1.1.

Fragment	Data subset	Substitution model
16S	n/a	GTR+G
COI	position 1	HKY+G
	position 2	F81+I
	position 3	HKY+G
CytB	position 1	HKY+G
	position 2	F81+I
	position 3	HKY+G
Wg	position 1	JC+I
	position 2	JC+I
	position 3	HKY+G

We conducted an additional analysis in which we combined the information from all gene trees into a single tree (species tree), since data from multiple genes and multiple individuals per species can be useful for resolving species trees [31–32]. We used *BEAST v1.8.0 [33] to infer species tree. The recognized five species and *S*. *septentrionalis* samples from AF were assigned as ‘‘species” in the analysis. The *BEAST run consisted of 50 million generations, a Yule process for the species tree prior, a piecewise linear and constant root model for population size, randomly generated starting trees for each gene, and a burn-in of 20%.

### Divergence time estimation

We inferred divergence times under a Bayesian framework using BEAST v.1.8.0 [34]. We generated the input file in BEAUTi using the two mitochondrial protein-coding genes (COI + CytB) and the substitution model (GTR + Γ) as selected by PartitionFinder. Only nucleotide data from ten specimens were included in these analyses in order to avoid missing data. We employed an uncorrelated lognormal relaxed clock model [35]. Clock models were unlinked, and substitution and tree models were linked among partitions. A Yule speciation process with a random starting tree was used for the tree prior. Given the poorly known fossil record for social wasps in general, with none belonging to *Synoeca* [36], we applied the Brower [37] mutation rate of mitochondrial genes (under a normal distributed prior). This mutation rate estimated at 2.3% My^-1^ was based on a set of seven studies that provided age estimates of lineage splits ranging from 300 to 3,250,000 years ago. Two independent Markov chain Monte Carlo (MCMC) searches were conducted with 100 million generations each, with parameters sampled every 10,000 steps and a burn-in of 20%. We checked for convergence between runs and analysis performance with Tracer v1.5 using effective sample size (ESS) scores. The resulting trees were combined using TreeAnnotator v1.8.0 and the consensus tree with the divergence times was visualized in FigTree v1.4.0.

### Historical biogeographic analysis

We performed the Bayesian Binary MCMC (BBM) method of biogeographical and ancestral state reconstruction implemented in RASP (Reconstruct Ancestral State in Phylogenies) 2.1b [38]. We used the tree obtained from *BEAST (species tree) and published occurrence data for the analyzed species [9] as input files for RASP. Thus, we assigned species distribution areas to geographical regions as follows: (A) Amazonian forest, (B) Middle America, (C) Atlantic forest, (D) Dry Diagonal (Cerrado, Chaco and Caatinga). The BBM analysis was run applying the model F81 + Γ and no outgroup was defined. We ran the analysis for 5 millions generations, sampled every 1000 generations with the first 1000 samples being discarded as burn-in.

## Results

### Morphological observations

We observed the presence of much punctation on the head of *Synoeca septentrionalis* and *S*. *chalibea*, in contrast to *S*. *virginea*, *S*. *cyanea*, and *S*. *surinama*, which showed little or no punctation on the head (Fig. 2a, b). Furthermore, *S*. *septentrionalis* have many erect setae on the scape (Fig. 2d), as opposed to *S*. *virginea*, *S*. *chalibea*, and *S*. *cyanea*, which do not present erect setae (Fig. 2c); *S*. *surinama* have few erect hairs on the scape. Another noteworthy morphological character is the presence of erect setae on the scutum of *S*. *septentrionalis*, *S*. *cyanea*, and *S*. *chalibea* that are absent from *S*. *surinama* and *S*. *virginea* (Fig. 2e, f). Moreover, the two *S*. *chalibea* samples studied here showed differences in body color, one form with a dark body (“dark form”) and the other form with the body entirely yellowish (“yellow form”).

### Molecular phylogeny

**Fig 3:**
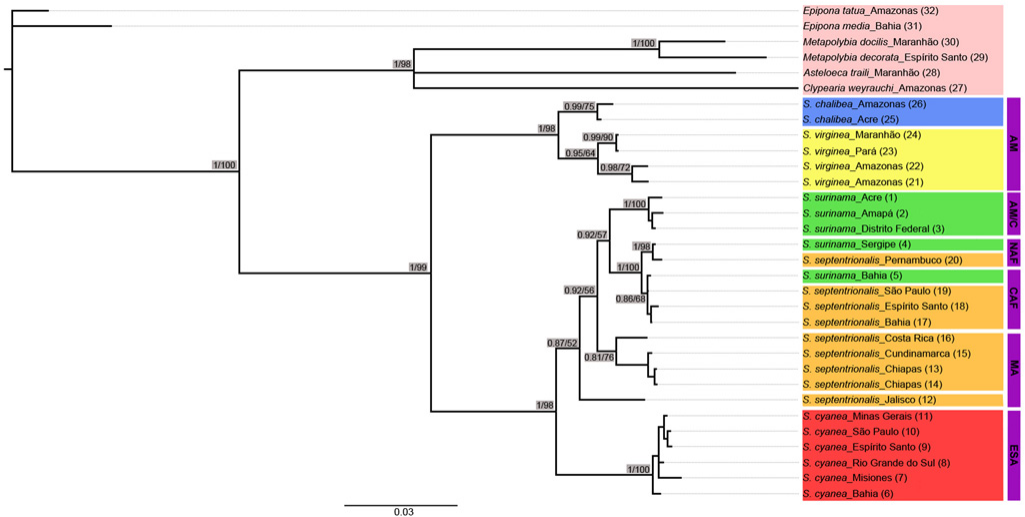
Phylogeny of *Synoeca* reconstructed by Bayesian analysis. Shown is the consensus tree resulting from analyses based on 1829 bp from 16S, CytB, COI and wingless. Maximum likelihood (ML) analysis resulted in the same topology. Numbers above or below branches indicate Bayesian posterior probabilities (first number) and ML bootstrap values (second number). Numbers in parentheses indicate the collection sites as shown in Fig. 1. Abbreviations for biogeographical units are as follows: AM, Amazonian forest; AM/C, Amazonian forest and Cerrado; NAF, North Atlantic forest; CAF, Central Atlantic forest; MA, Middle America; ESA, Eastern portion of South America.

Our concatenated data matrix of 1829 aligned nucleotide sites contained 416 variable sites, including 521 aligned base pairs (bp) of 16S (28.5% of data set), 472 bp of COI (25.8%), 433 bp of Cytb (23.7%), and 403 bp of Wg (22%). The BI and ML results from this concatenated data matrix are summarized in Fig. 3. The two types of analyses inferred the same topology with differences only in the degree of support for some nodes. Monophyly of the genus *Synoeca* is supported with high support [posterior probability (PP): 1 and bootstrap (BS): 0.99; Fig. 3]. The genus is divided into two major clades with high node support (PP: 1 and BS: 0.99; Fig. 3) containing: (1) *S*. *chalibea* and *S*. *virginea*; and (2) *S*. *cyanea*, *S*. *septentrionalis*, and *S*. *surinama*. Within the latter clade, *S*. *cyanea* is sister to *S*. *septentrionalis* and *S*. *surinama* with weak support (PP: 0.87 and BS: 0.52; Fig. 3). *Synoeca septentrionalis* samples from Middle America [henceforth called MA denoting samples from the Central America Rainforest region (Costa Rica), Southern North America (Mexico) and Northwestern South America (Colombia)] do not cluster with *S*. *septentrionalis* samples from AF. *S*. *surinama* samples from Acre, Amapá, and Distrito Federal (this group henceforth called AM/C denoting samples from both Amazon forest and Cerrado) do not cluster with *S*. *surinama* from AF. Moreover, *S*. *surinama* samples from the AF formed distinct clusters with *S*. *septentrionalis* from AF as following: the *S*. *surinama* sample from Bahia clusters with *S*. *septentrionalis* samples from Bahia, Espírito Santo, and São Paulo (this group henceforth called CAF denoting samples from Central Atlantic forest); the *S*. *surinama* sample from Sergipe clusters with *S*. *septentrionalis* sample from Pernambuco (this group henceforth called NAF denoting samples from the North Atlantic forest) (Fig. 3).

**Fig 4:**
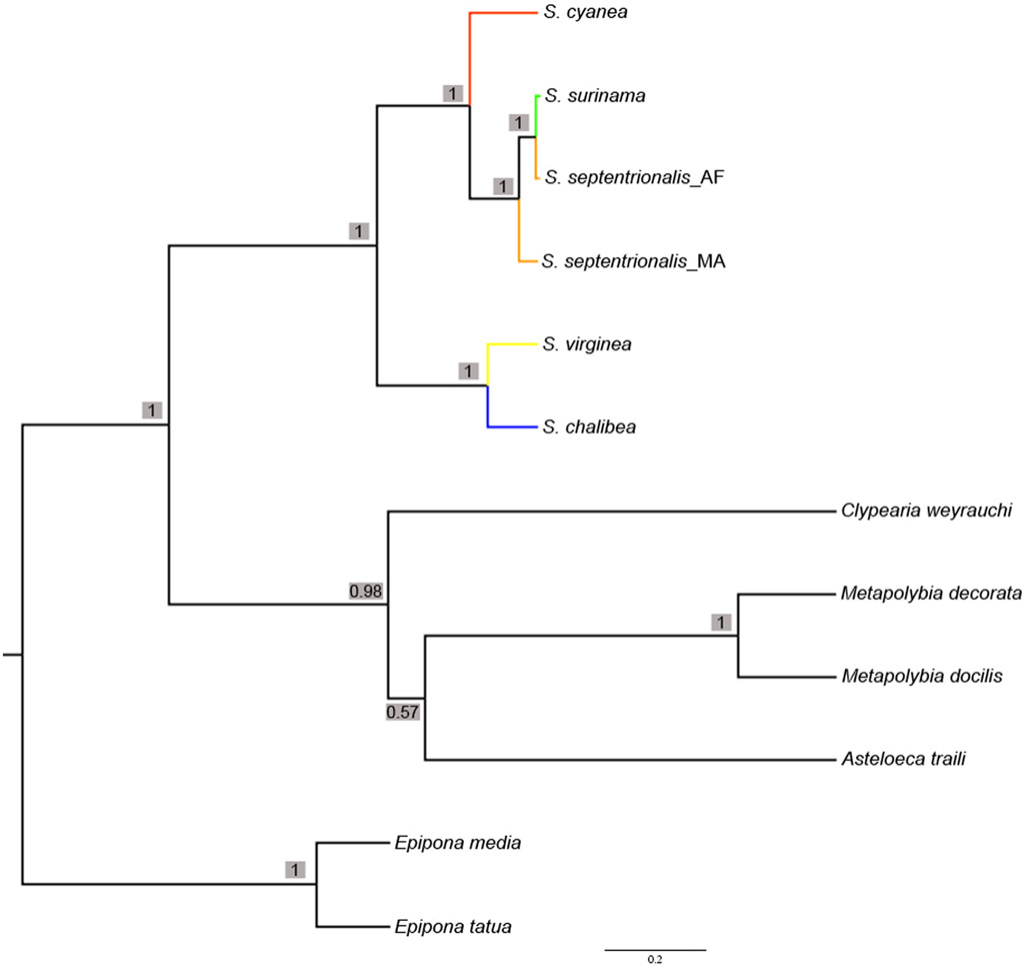
Species tree generated by *BEAST. Bayesian posterior probabilities indicated above or below branches. Branch colors follow Fig. 3.

BI performed on each of the three mitochondrial gene fragments all show divergence into the two major clades in *Synoeca* and consistently place *S*. *surinama* into the three groups discussed above, but there are some potential conflicts regarding the placement of some *S*. *septentrionalis* specimens among the three gene fragments (S1 Fig.). The species tree generated by *BEAST was congruent with the concatenated BI and ML results with higher node support (PP: 1) for all ingroup clades (Fig. 4).

### Divergence times and historical biogeography

**Fig 5:**
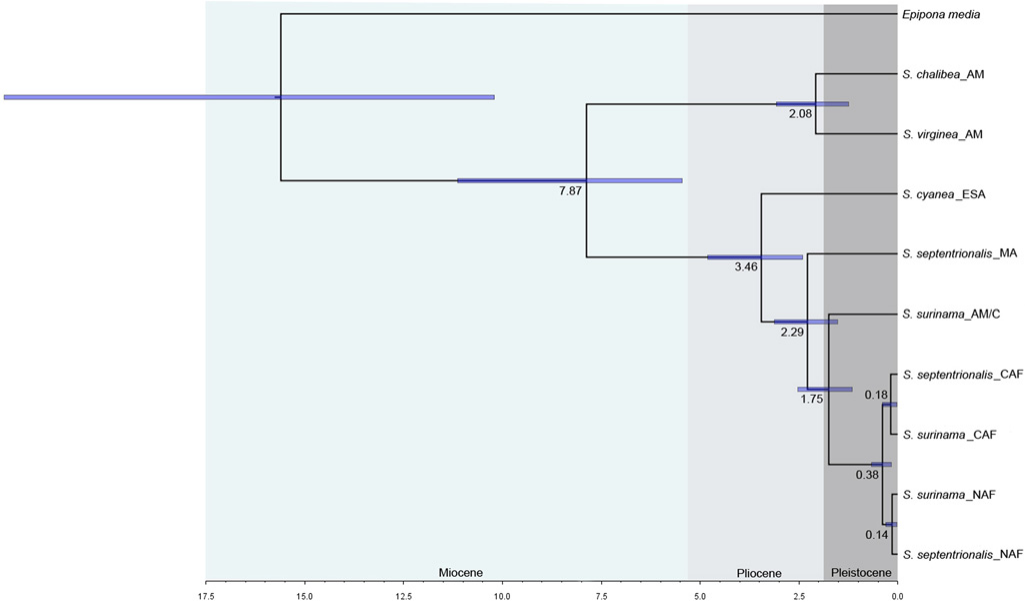
Chronogram with divergence times generated by BEAST. Divergence times between clades are annotated on each node and standard deviations [95% of high posterior density (HPD)] are represented by the blue bars.

**Fig 6:**
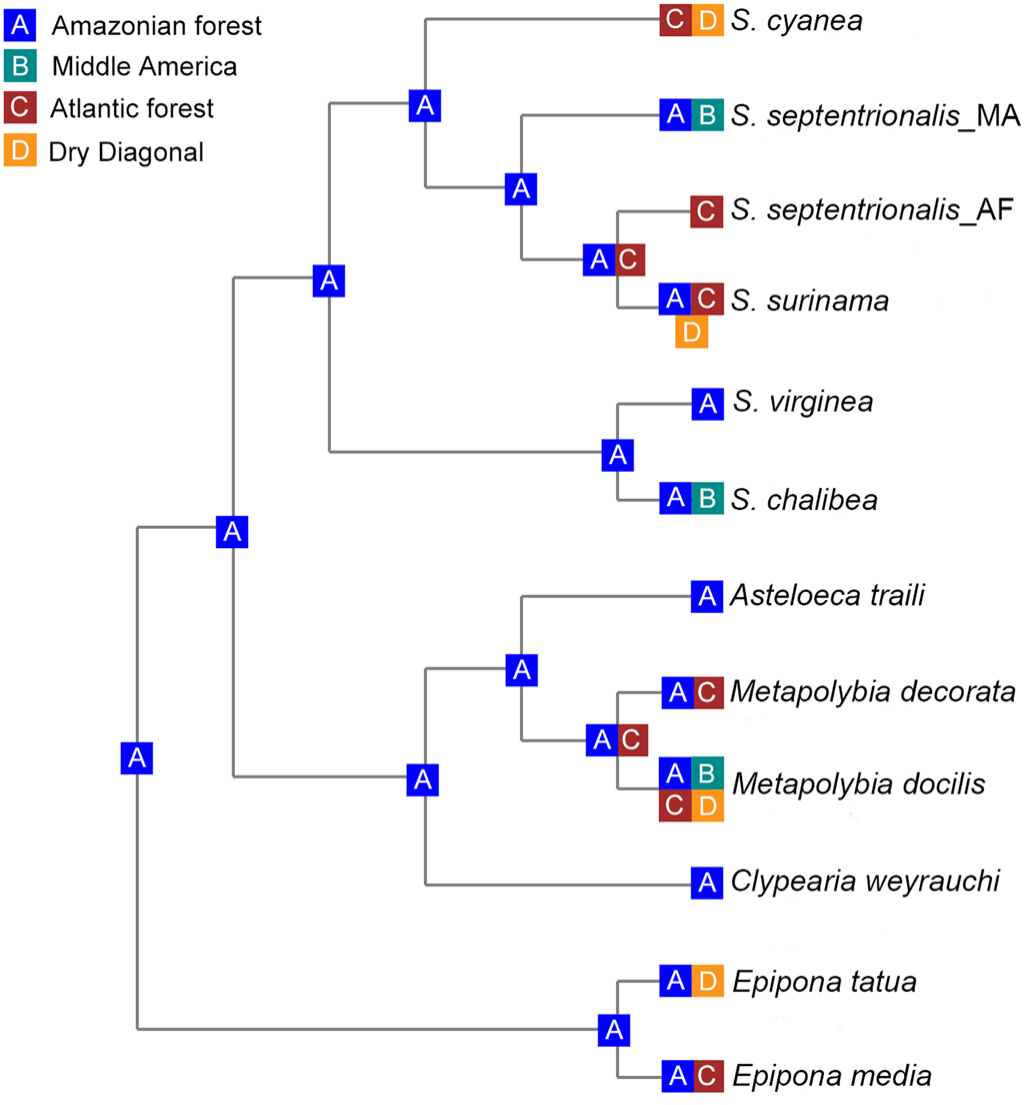
Biogeographical analysis using Bayesian Binary MCMC, showing only the most likely states at each node.

The divergence dating analysis revealed a middle/late Miocene origin for *Synoeca* with subsequent diversification of extant species occurring in the Plio-Pleistocene (Fig. 5). The oldest divergence event was the split between the two major clades: (*S*. *chalibea* + *S*. *virginea*) and (*S*. *cyanea* + (*S*. *septentrionalis* MA + *S*. *surinama/ S*. *septentrionalis* AF)) at 7.87 million of years ago (mya) [95% of high posterior density (HPD): 11.16–5.44 mya]. The youngest diversification event was the separation of specimens of *S*. *surinama* and *S*. *septentrionalis* in the northern clade at 0.14 mya (95% of HPD: 0.02–0.31 mya). The biogeographic ancestral area analysis supports an Amazonian origin for *Synoeca*, with three independent dispersal-vicariance events from Amazonia to the Dry Diagonal and AF, one secondarily from Amazonia to Central America, and one from Amazonia to the AF (Fig. 6).

## Discussion

### *Synoeca* systematics

Despite the fact that *Synoeca* has only five species described, often some specimens are misidentified in collections. This problem was pointed out by Richards [9], for example concerning the misidentification of specimens of *S*. *chalibea* and *S*. *virginea*, which requires careful comparison. Indeed, the “yellow form” of *S*. *chalibea* is quite similar to *S*. *virginea*, but can be differentiated by the presence of punctation on the scutum and pronotum [20], as well as on the head. Also, specimens of *S*. *septentrionalis* from the AF show variation in the dark triangular area in the clypeus as verified by Menezes et al. [19], a major character used in the diagnosis of this species according to Cely and Sarmiento [21]. In some specimens, this dark area is totally absent, which may cause this species to be misidentified as *S*. *cyanea*, which also has a reddish clypeus. Considering this, specimens from AF identified as *S*. *cyanea* in collections need to be carefully verified. Despite the morphological similarities between *S*. *septentrionalis* AF and *S*. *cyanea*, the new morphological characters described here can be useful for correct species identification.

The division of *Synoeca* into two major clades is in disagreement with previous morphological phylogenetic analyses [20, 22]. However, morphological characters, such as punctation on the propodeum, clypeal-eye contact, malar space and wing color, might separate *Synoeca* into two groups (see [9, 20]) as shown here: (*S*. *chalibea* + *S*. *virginea*) + (*S*. *cyanea* + (*S*. *surinama*/*S*. *septentrionalis*)). Moreover, the previous placement of *S*. *septentrionalis* as sister to the clade (*S*. *surinama* + *S*. *cyanea*) is not supported by our molecular results. This clade in the morphological analyses is supported only by the absence of numerous erect outstanding setae on the first metasomal tergum and sternum (see [20]). However, Andena et al. [20] commented that this condition is homoplastic because it is found in several outgroups. Thus, this character may not be sufficient to establish relationships between the species within the clade *S*. *cyanea* + *S*. *surinama*/*S*. *septentrionalis*.

### Historical biogeography

Our results also shed light on biogeographic patterns within *Synoeca*. Three *Synoeca* species occur in the AM (*S*. *chalibea*, *S*. *virginea* and *S*. *surinama*), *S*. *septentrionalis* occurs in the MA and AF, and only one species is restricted to the ESA, namely *S*. *cyanea*. Also, most of the species of the tribe to which *Synoeca* belongs (Epiponini) occurs in or is restricted to the AM [9]. Moreover, our analysis of ancestral area reconstruction supports an Amazonian origin of *Synoeca* (Fig. 6). Thus, the genus may have experienced three main colonization events from Amazonia during the Plio-Pleistocene.

The oldest inferred route probably occurred in southern South America between the AM and the AF. There is much evidence to support contact in the past between the AF and the AM [6, 7, 39–41]. Batalha-Filho et al. [7] combined phylogenetic and distributional data of avifauna and suggested old connections (middle to late Miocene) between AM and AF through the current southern Cerrado and Pantanal and the transition towards the Chaco and palm savannas of Bolivia and Paraguay. Also, it has been suggested that taxa representing divergences through this connection in the AF are spatially restricted to the southern AF and upland forests in southern Bahia, Minas Gerais, Espírito Santo, Rio de Janeiro and São Paulo [7]. Thus, the current geographical distribution of *S*. *cyanea* and its time of diversification at 3.46 mya (95% of HPD: 4.79–2.4 mya; Fig. 5) are consistent with this species colonizing by this route in southern South America.

A second route probably occurred relatively recently in direction to the Central America Rainforest region and southern North America via the Isthmus of Panama. The formation of Isthmus of Panama during the Pliocene at ~3.5 mya led to the Great American Biotic Interchange [2, 42]. The current distribution and diversification time of *S*. *septentrionalis* MA at 2.29 mya (95% of HPD: 3.15–1.56 mya; Fig. 5) agrees with this colonization route. Moreover, the lower species richness of Epiponini in the southern North America and Central America Rainforest regions compared to South America may be explained by recent dispersal via the Isthmus of Panama and, therefore, less time for species diversification.

The third route appears to have recently occurred in northeastern Brazil between AM and AF. Batalha-Filho et al. [7] suggest two connection pathways between AM and AF in northeastern Brazil, one through the coastal zones of Maranhão, Piauí, Ceará, and Rio Grande do Norte (Brazil), and another through Tocantins and Bahia (Brazil). Populations of *S*. *surinama* may have reached the AF by one of these routes and, after the breaking of this connection, experiencing consequent divergence between populations of AM/C and AF. Genetic differences among populations in both AM and AF are also found in the swarm-founding social wasp *Angiopolybia pallens* [43].

### Atlantic Forest and *S*. *septentrionalis*

Despite the morphological similarity seen in *S*. *septentrionalis* specimens living in different regions, our molecular data suggest that *S*. *septentrionalis* and *S*. *surinama* specimens from the Atlantic Forest (AF) are interrelated and belong to a distinct lineage of *Synoeca*. Moreover, there appears to be a very recent division [at 0.38 mya (95% of HPD: 0.67–0.16 mya; Fig. 5)] between northern and central Atlantic forest groups (NAF and CAF). This division may be explained by the occurrence of refuges during the Pleistocene in the northern and central AF seen by Carnaval et al. [44] for amphibians. However, our molecular results may also be due to incomplete lineage sorting and/or mitochondrial introgression between *S*. *septentrionalis* AF and *S*. *surinama* AF. Nevertheless, there is insufficient morphological information to separate *S*. *septentrionalis* AF and *S*. *septentrionalis* MA and we suggest that they may represent two species with a very similar morphology (i.e., potential cryptic species). Further studies at the population level will be useful in characterizing the diversification processes occurring between members of *S*. *surinama* and *S*. *septentrionalis*.

Our molecular phylogenetic findings suggest that *Synoeca* species richness in the Neotropical Region may be underestimated due to morphological similarity and lack of broad geographical sampling. Further studies combining morphology, genetics, and population-level sampling should be seen as the main challenge in the future of phylogenetic research in social wasps as a whole. This work may result in the future recognition of additional species of social wasps in the Neotropics.

## Supporting Information

S1 Fig Trees constructed by Bayesian inference for each mitochondrial gene. (a) 16S, (b) COI and (c) CytB.
(TIF)

S1 Table Target gene fragments, primer sequences, and origin of the primers used in this study. (DOCX)
