# Supplementary figures and images for: Molecular Phylogeny and Historical Biogeography of the Neotropical Swarm-Founding Social Wasp Genus Synoeca (Hymenoptera: Vespidae)
Source: PLoS One. 2015 Mar 4;10(3):e0119151. doi: 10.1371/journal.pone.0119151 (PMC4349807; doi:10.1371/journal.pone.0119151)

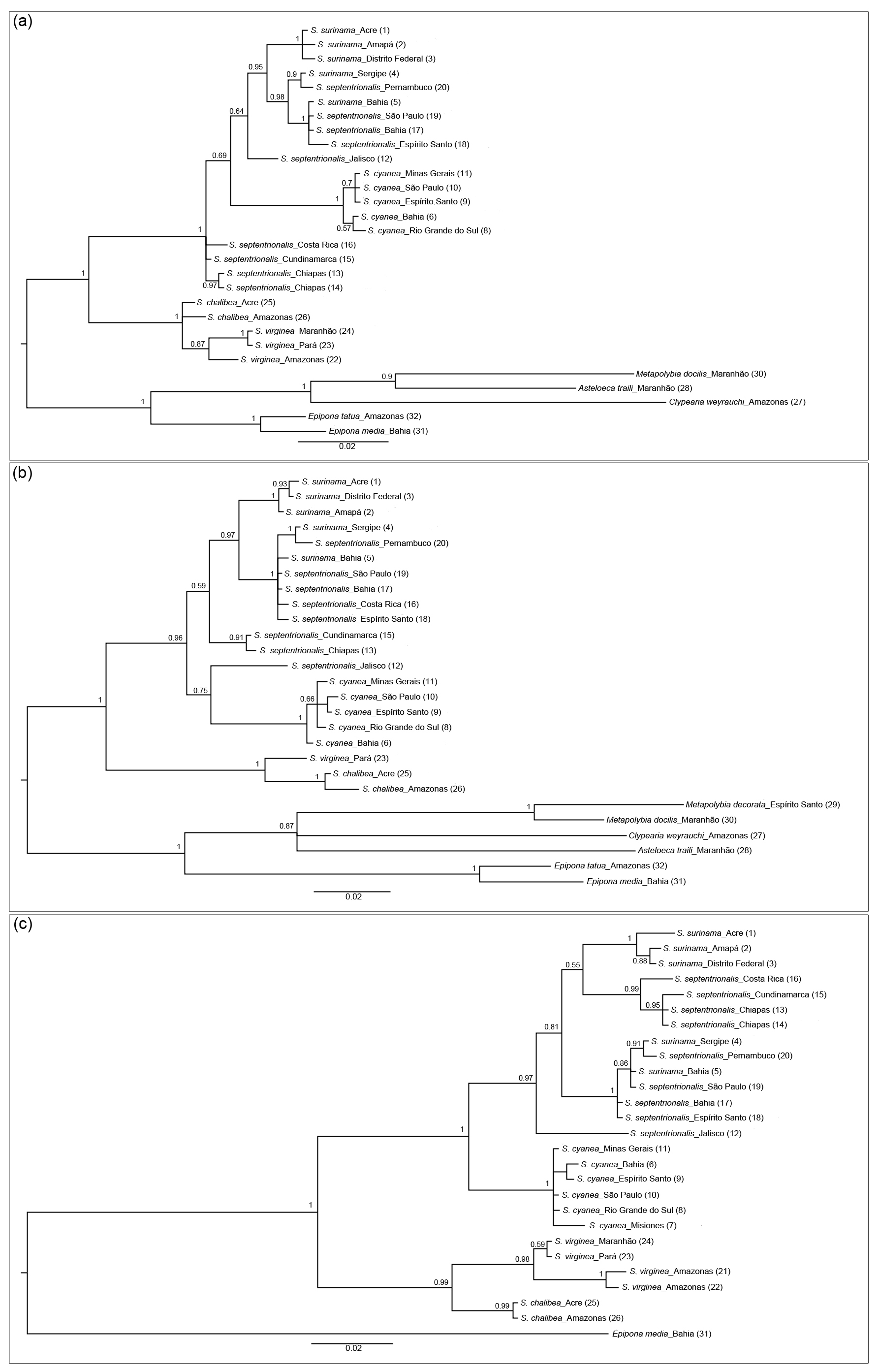

Supplement: S1 Fig — (a) 16S, (b) COI and (c) CytB. (TIF) [file pone.0119151.s001.tif]
