# Supplementary material for: Molecular Phylogeny and Historical Biogeography of the Neotropical Swarm-Founding Social Wasp Genus Synoeca (Hymenoptera: Vespidae)
Source: PLoS One. 2015 Mar 4;10(3):e0119151. doi: 10.1371/journal.pone.0119151 (PMC4349807; doi:10.1371/journal.pone.0119151)
Supplement: S1 Table — (DOCX) [file pone.0119151.s002.docx]

| Fragment | Primers | Source |
| --- | --- | --- |
| Cytochrome b | CYTB – CRO91A: 5´- TATGTACTACCATGAGGACAAATATC – 3´ | Crozier et al., 1993 |
|  | CYTB – CRO91B: 5´- ATTACACCTCCTAATTTATTAGGAAT – 3´ | Crozier et al. 1993 |
| 16S | LR13943F: 5` - CACCTGTTTATCAAAAACAT - 3` | Costa et al., 2003 |
|  | LR13392R: 5` - CGTCGATTTGAACTCAAATC – 3` | Costa et al., 2003 |
| COI | F: CI-J-1718: 5` - GGAGGATTTGGAAATTGATTAGTTCC– 3` | Simon et al., 1994 |
|  | R: CI-N-2191: 5` - GGTAAAATTAAAATATAAACTTC – 3` | Simon et al., 1994; Kambhampati and Smith, 1995 |
| wingless | Wg578F:  TGCACNGTGAARACYTGCTGGATGCG | Ward and Downie, 2005 |
|  | Wg1032R:  ACYTCGCAGCACCARTGGAA | Abouheif and Wray, 2002 |

**References**

Abouheif E, Wray GA (2002) Evolution of the gene network underlying wing polyphenism in ants. Science (Washington D.C.) 297: 249-252.

Costa MA, Del Lama MA, Melo GAR, Sheppard WS (2003) Molecular phylogeny of the stingless bees (Apidae, Apinae, Meliponini) inferred from mitochondrial 16S rDNA sequences. Apidologie 34: 73–84.2.

Crozier RH, Crozier YC (1993) The mitochondrial genome of the honeybee *Apis* *mellifera*: complete sequence and genome organization. Genetics 133: 97-117.

Kambhampati S, Smith PT (1995) PCR primers for the amplification of four insect mitochondrial gene fragments. Insect Mol Biol 4: 233-236.

Simon C, Frati F, Bechenbach A, Crespi B, Liu H, et al. (1994) Evolution, weighting, and phylogenetic utility of mitochondrial gene sequence and compilation of conserved polymerase chain reaction primers. Ann Entomol Soc Am 87: 651-701.

Ward PS, Downie DA (2005) The ant subfamily Pseudomyrmecinae (Hymenoptera: Formicidae): phylogeny and evolution of big-eyed arboreal ants. Syst Entomol 30: 310-335.
